# An exceptionally large coronary artery aneurysm in a formerly healthy young woman

**DOI:** 10.1007/s12471-015-0756-8

**Published:** 2015-10-08

**Authors:** B.C. Du Pré, L.W. Van Laake, B.K. Velthuis, E.E.C. de Waal, M.P. Buijsrogge, R.J. Hassink

**Affiliations:** 1 Department of Cardiology, University Medical Center Utrecht, Heidelberglaan 100, 3584 CX Utrecht, The Netherlands; 2 Department of Medical Physiology, University Medical Center Utrecht, Utrecht, The Netherlands; 3 Department of Radiology, University Medical Center, Utrecht, The Netherlands; 4 Department of Anesthesiology, University Medical Center Utrecht, Utrecht, The Netherlands; 5 Department of Cardiothoracic Surgery, University Medical Center Utrecht, Utrecht, The Netherlands

A 32-year-old woman presented with progressive dyspnoea for 6 weeks, fatigue, weight loss, chest pain, and night sweats. Apart from an uncomplicated delivery of her third child 4 months ago, she had no medical or family history. Pneumonia was suspected (chest X-ray Supplementary Fig.1a, b), but thoracic computed tomography (CT) was performed to exclude pulmonary emboli.

The CT surprisingly showed a large intrathoracic mass of 94 × 80 mm extending from the sternum to the thoracic spine, compressing the superior and inferior caval veins, the right atrium, and (partly) the right ventricle (Fig. 1, Supplementary Fig. c–e, and Video 1). Further analysis revealed a giant coronary aneurysm (GCA) originating from the proximal right coronary artery (RCA) connecting inferiorly to the distal RCA. Left ventricular function was good (Video 2 and 3). Due to the size and the location of the GCA, surgical exclusion was the treatment of choice (Fig. 2, Supplementary Fig. F). After surgery, the patient recovered well and was discharged on the fifth postoperative day.

**Fig. 1:**
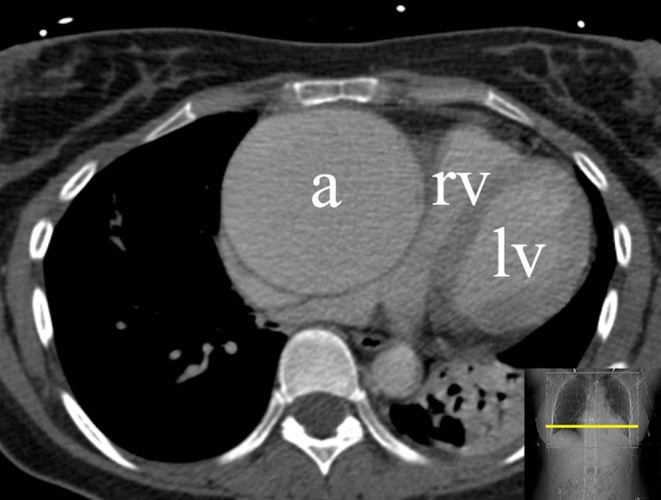
Thoracic CT showing a large intrathoracic mass. Note the equal densities of the aneurysm and the left ventricular lumen

**Fig. 2:**
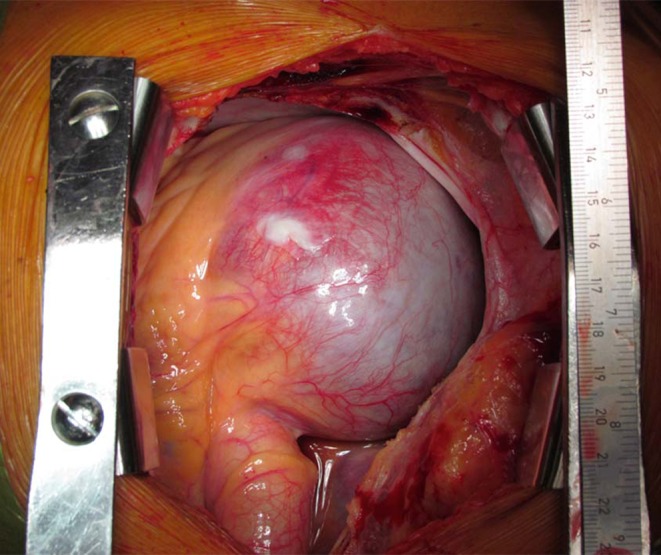
Giant coronary aneurysm after sternotomy

GCAs are extremely rare: less than 0.02 % of all cardiac surgery is attributed to GCAs [1]. They are usually related to comorbidities or injuries such as infectious disease, inflammatory disease, trauma, coronary angioplasty, or connective tissue disease but can also occur as a congenital abnormality. [4] It is likely that in the current case, the GCA had existed for many years. Increased workload and hormonal changes during pregnancy and delivery may have contributed to growth and symptoms of the GCA [2, 3].

## Conflict of interest

None declared

## Electronic supplementary material

(JPG 318 kb)

(WMV 1271 kb)

(AVI 759 kb)

(AVI 431 kb)
