# Supplementary figures and images for: An exceptionally large coronary artery aneurysm in a formerly healthy young woman
Source: Neth Heart J. 2015 Oct 8;23(12):609–10. doi: 10.1007/s12471-015-0756-8 (PMC4651965; doi:10.1007/s12471-015-0756-8)

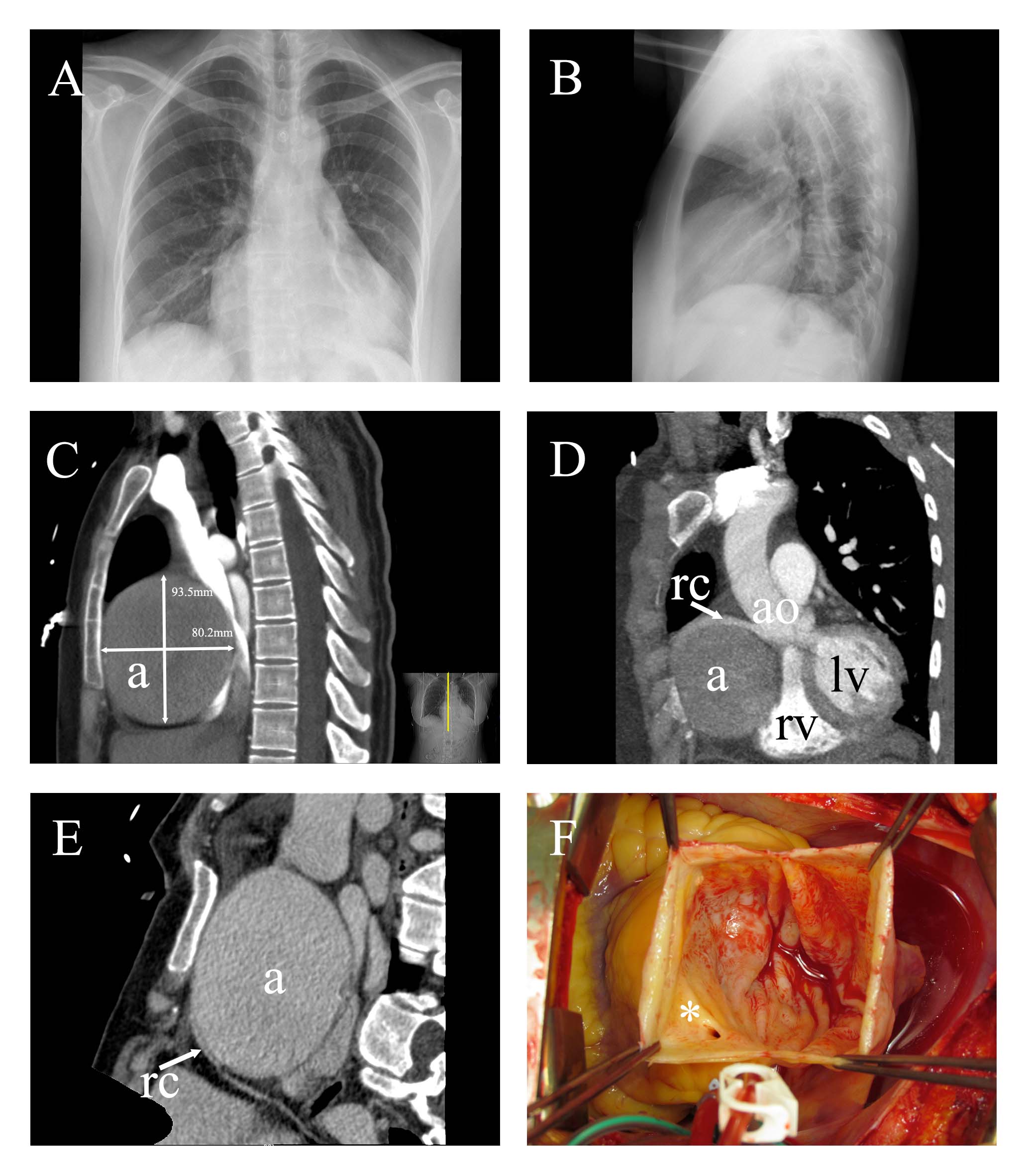

Supplement: Supplementary file 1 — (JPG 318 kb) [file 12471_2015_756_MOESM1_ESM.jpg]
